# HPV vaccine: an overview of immune response, clinical protection, and new approaches for the future

**DOI:** 10.1186/1479-5876-8-105

**Published:** 2010-10-27

**Authors:** Luciano Mariani, Aldo Venuti

**Affiliations:** 1 Dept. Gynaecologic Oncology, National Cancer Institute Regina Elena of Rome, Italy; 2 Lab. Virology, National Cancer Institute Regina Elena of Rome, Italy

## Abstract

Although long-term protection is a key-point in evaluating HPV-vaccine over time, there is currently inadequate information on the duration of HPV vaccine-induced immunity and on the mechanisms related to the activation of immune-memory. Longer-term surveillance in a vaccinated population is needed to identify waning immunity, evaluating any requirements for booster immunizations to assess vaccine efficacy against HPV-diseases. Current prophylactic vaccines have the primary end-points to protect against HPV-16 and 18, the genotypes more associated to cervical cancer worldwide. Nevertheless, data from many countries demonstrate the presence, at significant levels, of HPVs that are not included in the currently available vaccine preparations, indicating that these vaccines could be less effective in a particular area of the world. The development of vaccines covering a larger number of HPVs presents the most complex challenge for the future. Therefore, long term immunization and cross-protection of HPV vaccines will be discussed in light of new approaches for the future.

## Introduction

The nature of antibody responses and duration, following HPV vaccination, plays a key role in long-term protection against papillomavirus infection. The importance of vigorous and prolonged immune protection over time is related to the following issues:

1. the risk of HPV-infection remains as long as women remain sexually active (at least 70-80% of risk during their lifetime); the rate of prevalence and incidence of high-risk HPV-infection is well documented in women over 26 yrs [1,2]. Furthermore, a population-based cohort study in Costa Rica showed that type-specific persistence increases with age [3]. All the above factors point-out that sexually active women over 25 are still at risk of acquiring a new HPV infection [4].

2. it is crucial to test the utility of HPV vaccination programs as public health interventions;

3. it displays the maximum benefits of cervical cancer and other HPV-related cancers.

Nevertheless, it should also be highlighted that long-term protection is not fully predictable at the introduction of any vaccine, because it varies according to many variables (cohort target, coverage, acceptance, catch-up...), that are not strictly related to immune response only. Although some authors have developed a model to predict long-term immunity, it still remains an ongoing and challenging issue, as well as other human vaccines: such as hepatitis B, meningococcal C or pneumococcal polysaccharide vaccines [5,6].

To better analyze this problem three main aspects will be valued: natural stability of HPV over time, immune response after natural HPV infection and after vaccine.

Finally, HPVs are a family of many different genotypes and ideally, an ideal vaccine should cover at least the majority of those linked to tumor development, the so called "high risk" types. Data from different Asian areas have pointed out that a significant number of pre- and neoplastic cervical lesions are linked to types 52 and 58 for example, which are rarely detected in Western countries. Thus, the possibility to develop second generation cross-reacting vaccines covering a larger number of HPVs will also be discussed.

## 1-Natural Stability of HPV

HPV genotype variation over time in cervical cancer is a crucial factor in estimating the long-term impact of vaccines. Indeed, papillomaviruses are an ideal model system for studying the DNA virus evolution. From our understanding of phylogenetic studies, the root of the evolution of HPV types should point to Africa, since humans evolved from nonhuman primates in this continent and where the separation of the a-, b- and g-PVs must have predated the origins of primates [7]. In addition, the phylogeny of HPV variants (3 lineages: European, Asian American and African) reflects the migration patterns of *Homo sapiens*. Although Xi et al. provided evidence that the evolutionary process stemmed from greater adaptability of certain intratype HPV variants to specific human population groups, HPVs have remained stable viruses over time, with unexpected major variations [8]. HPVs do not change host species and do not reorganize themselves. They have maintained their basic genomic organization for a period exceeding the 100 million year period. Furthermore, the spectrum of diseases associated with HPV infections [anogenital cancer and warty lesions], have accompanied humans throughout evolution.

Therefore, differently from the quasi-species of many RNA viruses, HPV types have evolved very slowly, and have diverged since the origin of humanity only by about 2% [9].

Over the next few decades the efficacy of a papillomavirus vaccine is fundamentally predictable. Nevertheless, the large number of different genotypes among the HPV viruses questions the number of HPV viruses that must be included in the vaccine preparation process.

## 2-Natural Immune response to HPV Infection

In biological evolution, HPVs are successful infectious agents. They induce persistent infections without frequent and serious complications for the host and shed virions for transmission to other naive individuals. They reach a balanced state where the host *usually *is not *seriously *disadvantaged by the HPV infection, and the virus is not too limited in reproducing by the host's immune response [10]. To achieve this lifestyle and to maintain a state of equilibrium, the HPV must avoid the host's defense systems. Many factors contribute to evading immune pools, in particular:

1. no viral-induced cytolysis or necrosis;

2. no inflammation;

3. little or no release into the local milieu of proinflammatory cytokines;

4. no blood-borne or viremic phase;

5. only minimal amounts of replicating virus exposed to immune defenses;

6. infection is exclusively intraepithelial;

7. virus capsid entry is usually an activating signal for DCs, but there is evidence that LCs are not activated by the uptake of HPV capsids;

8. free virus particles are shed from the surface of squamous epithelia with poor access to vascular and lymphatic channels and to lymph nodes where immune responses are initiated;

9. most DNA viruses have mechanisms for inhibiting interferon synthesis and receptor signaling, and papillomaviruses are no exception.

In fact, as quoted by Mark H Einstein "...these escape mechanisms have enabled HPV to become one of the most common sexually transmitted infections worldwide" [11].

Despite HPV's ability to evade the host's immune system and to down-regulate innate immunity, a primary HPV-infection is cleared naturally in approximately 90% of cases, thus indicating the central role of immunity in the resolution of cervical and anogenital HPV-associated diseases. Conversely, clearance of papillomavirus infection is significantly impaired in women with HIV/AIDS or in immunosuppressed renal transplant patients, thus focusing on the importance of cell-mediated immune responses to HPV infection [12,13]. Without a doubt CD4+ T-helper cells are almost certainly crucial in avoiding persistent HPV infection, as well as inducing wart regression [14,15].

The host's immune response to HPV infection (humoral immunity, mainly IgG) is usually slow, weak and varied considerably among women. Generally, close to half of the individuals seroconverted to L1 protein of HPV-16, -18, or -6 within 18 months [16]. Conversely, it means that more than 40% of women do not seroconvert or wane over time from the immune response and therefore, indicates that the HPV L1 capsid-specific antibody is not a suitable diagnostic test for HPV infection. Other HPV antigens [E1, E2, E6, and L2] do not evoke any antibody responses in patients with acute or persistent HPV infection.

Innate immunity acts as the first line of nonspecific defense against any pathogen (dendritic cells, interferon-α, cytokines, neutrophils, and macrophages) and attacks by HPV should be detected by the intraepithelial dendritic cell (DC). There is evidence indicating that DCs are not activated by the uptake of HPV capsids suggesting a limited role in the host's response to HPV infection [17,18].

In regards to the differences versus post-vaccination immunity, it should stress two main critical points. The first is the possible immunodominant nature of the immune response after natural infection. Most antigens are structurally complex, containing many different epitopes or antigenic determinants. The L1 capsid protein contains multiple overlapping epitopes, some of which may be immunodominant. The immune system responds to the antigen by producing a higher rate of neutralizing antibodies to the most accessible epitopes or to the immunodominant types. However, in natural HPV infection the immune response is weak and type-specific. Conversely, after administrating the HPV vaccine a strong and, although partially, cross-reactive immune-response was detected.

The second critical point is in regards to the long-term clinical significance of immunity evoked by natural infection. Some clinical studies suggest that natural infection-elicited antibodies may not provide complete protection to HPV over time. However, they could not distinguish the new infection from the reactivated latent ones. Recurring HPV type specific natural infections occur equally in women after 5-7 years of follow-up, regardless of the type specific serostatus [19]. Similar indications emerge from the quadrivalent vaccination trial. It has been established in the FUTURE studies that some women in the placebo-group developed the disease despite having antibodies to the offending HPV types at enrollment, thus confirming, as stated in the recent WHO position-paper, that host antibodies, mostly directed against the viral L1 protein, "...*do not necessarily protect against subsequent infection by the same HPV genotype"*[20,21].

## 3-Immune response to HPV Vaccine

The most effective HPV vaccine was developed as a result of the achievement of core technologies able to produce virus-like particles (VLPs). The recombinant DNA was used to generate VLPs capable of mimicking the natural virus and eliciting high-titers of virus neutralizing antibodies. The L1 gene from the viral genome was sub-cloned in microorganisms, such as yeast (for quadrivalent vaccine) or baculovirus (for bivalent vaccine). In this way L1 over-expressed proteins spontaneously self-assemble into VLPs that:

1. resemble the conformation of authentic virions;

2. are neither infectious, nor oncogenic;

3. induce high levels of *type specific neutralizing *antibodies.

The main question about the HPV vaccine is:

### why, when the body's natural antibodies respond so poorly, do HPV-vaccines that generate serum neutralizing antibodies work?

The answer is that the quality and the quantity of the immune response generated by the vaccine is different to those of the natural infection.

The main characteristics of the immune response following VLPs are:

1. VLPs are highly immunogenic [two log over the natural infection], inducing high concentrations of neutralizing Ab to L1, also in the absence of adjuvant ones [due to their ability to activate both innate and adaptive immune responses] and they also remain high over time;

2. VLPs generate a heterogeneous or polyclonal immune response: immunodominant and non-immunodominat; neutralizing and non-neutralizing; type-specific and partially cross-reactive type responses.

3. The antigen dose in VLPs is much higher than in natural infection and the capsids are directly exposed to systemic immune responses.

A rapid, potent, and sustained immunologic response to the administration of a quadrivalent vaccine (targeting HPV 6, 11, 16, and 18) and after a bivalent vaccine (targeting HPV 16 and 18) has been reported so far [22,23]. Antibody titres (expressed as geometric mean titres -GMTs- of serum IgG) reach their peak after the third dose, then decline gradually, but remain higher than those naturally infected. Such high immune-responses mean high clinical protection [in the short-term evaluation of both trials], close to 100% in HPV-naïve women against CIN2+ or AIS [24-26].

Another question that we are faced with is: **does the intensity of such a humoral immune response correlate with long-term protection? **Although a direct correlation between antibody levels and protection may seem intuitively obvious, it is still unclear whether differing antibody titers indicate better disease protection or longer duration of immune protection [27].

Given that virtually all vaccinated women are seroconverted, we may deduce that up-to-now, we do not have any immunological correlates for protection as already stated in the last WHO position paper and therefore, the question still remains unanswered [21].

It was estimated that near life-long persistence of anti-HPV-16 and 18, following bivalent vaccination, is expected at titer levels above those associated with reduction of natural HPV-16 infection in 76% of these subjects, and above detectable levels in 99% of these subjects[6,28]. However, even in women where post-vaccine antibody levels drop to natural infection levels [such a humoral response against L1 HPV-18 in the quadrivalent RCT], there is no evidence to date of a vaccine breakthrough [29]. Indeed, in other infectious diseases [such as human hepatitis A and B] the persistence of immunity in individuals with decreasing antibody levels after vaccination has been demonstrated [30]. In addition, animal models show that low levels of anti-L1 antibodies provide long-term protection against high doses of the challenging virus [31]. As quoted by Margaret Stanley, published data on overall RCTs extend only to 5.5 years post immunization, therefore, the question of disease protection in the absence of detectable antibodies still remains [32]. However, recent data indicated that efficacy, immunogenicity, and safety of the bivalent AS04-adjuvanted vaccine is up to 6.4 years [33]. This period is the longest reported for any HPV vaccine suggesting that boosters are not needed later on, decreasing considerably the complexity and costs of the delivery programme, particularly in developing countries [34]. Nevertheless, as this is still a serious issue, the same bivalent vaccine HPV007 Study Group is carrying out a separate follow-up study continuing up to 9.5 years after vaccination in a subset of women from the previous study.

After having dealt with the latter issue, we must try and address the following question:

### Does the vaccine activate the immune-memory system?

In other words, is it stated that vaccines will induce a generation of long-life memory immune cells that, after re-exposure to the relevant antigen, generate a potent immune response preventing HPV infection?

The mechanisms of long-term immune-protection by means of memory B-cells have been, once again, elucidated for the hepatitis B virus vaccine, whose evoked-immunity appears similar to that of the HPV vaccination [35]. Certainly, memory B cells play an important role in effective immunization and in the memory-mechanism that produces antibodies in response to further antigenic challenges [36]. Indeed, circulating B memory cells can be detected soon after HPV bivalent vaccination [37]. Furthermore, the study of Einstein et al, comparing the immune response and reactogenicity of the two vaccines with the same methodology [PBNA, pseudovirion-based neutralization assay] stated that for any age strata positivity rates for anti-HPV-16 and -18 neutralizing antibodies in cervicovaginal secretions and circulating HPV-16 and -18 specific memory B-cell frequencies were higher after vaccination with the bivalent vaccine compared with the quadrivalent vaccine [38].

WHO explicitly stated that the induction of the immune memory "*should be assessed by means of evaluating immune responses to additional doses of vaccine administered at planned intervals following completion of the primary series*" [39]. Subsequently, the immune-memory anamnestic response with an antigen challenge has been reported for the quadrivalent vaccine [40]. Nevertheless, the question in vaccinated women is: does natural re-exposure to the same HPV type-vaccine significantly boost antibody levels, which contribute to the long-term persistence of anti-HPV responses and, consequently, does it improve protection over the next few decades? Time is needed to suitably answer this question.

## 4-Second Generation of Cross-Reacting Vaccines

The current vaccines are able to elicit an immunological response against the two most common oncogenic types found in cervical cancer, HPV-16 and HPV-18, but not against all high-risk mucosal HPVs. Data from many countries demonstrate the presence, at significant levels, of HPVs that are not included in the currently available vaccine preparations indicating that these vaccines could be less effective in certain areas of the world [41-43]. It is obvious that a multivalent vaccine against a multitude of HPVs will have a major impact on public health, and efforts to develop a nine-type L1 VLP combination vaccine are ongoing. Undeniably, VLP vaccines are highly effective against the virus types from which the L1 originates from, but their efficacy against other HPV types is variable, depending, in part, upon their phylogenetic similarity [44].

Preventing infection and disease associated with additional oncogenic genotypes, immunologically related to HPV 16 and 18 (particularly HPV 31 and 45), may provide an extra measure of protection. A statistically significant protective effect against virological and clinical end-points regarding HPV 31 (persistent infection and CIN2-3/AIS related diseases) has been reported after administrating the quadrivalent vaccine in the naïve [45] and ITT populations [46].

Also, after administrating the bivalent vaccine, a cross-protection against incident infection (with a 66 months of follow-up), persistent infection and CIN2+ related to HPV 31 and HPV-45 has been reported[47,26].

### L2 vaccines

Many reports suggested that immunization against the minor capsid protein 2 might work as a pan-HPV vaccine against different genotypes of papillomaviruses in addition to those causing genital warts and/or cervical and other mucosal cancers. Preclinical studies have demonstrated that cow or rabbit immunizations with L2 polypeptides protect against the homologous animal papillomavirus at mucosal sites in the bovine papillomavirus (BPV) type 4/cattle model and at cutaneous sites in the cottontail rabbit papillomavirus (CRPV)/rabbit model [48-53]. In addition to homologous protection, inoculation of amino-terminal L2 polypeptides also induced protection against heterologous papillomavirus types. Indeed, vaccination with HPV-16 L2 (amino acids 11-200) protects against CRPV and rabbit oral papillomavirus, both evolutionarily divergent from HPV-16 [54]. Vaccination with BPV-1 L2 (amino acids 1-88) peptides produced sera with cross-neutralizing activity against different HPVs [55]. Protection induced by homologous and heterologous L2 polypeptides, appears to be mediated by neutralizing antibodies. Human volunteers vaccinated with the candidate prophylactic/therapeutic vaccine HPV-16 L2E6E7, fusion protein produced L2-specific antibodies, neutralized a divergent type of HPV [39 ,56].

Thus, the efficacy of L2 vaccination has been proved in pre-clinical and clinical studies but, since natural infection does not induce anti-L2 antibodies [37] and many L2 epitopes are not on the virus surface [57], **how can the antibodies against the L2 N-terminal region neutralize the virus?**

A possible explanation is that during the infection cellular protease furin removes an L2 N-terminal sequence rendering L2 accessible on the capsid surface and displaying the L2-neutralizing epitopes. Thus, the binding of the anti-L2 antibodies to the exposed L2 epitope[s] blocks virus transfer from the extracellular matrix to the cell surface and hence prevents infection [58,59].

However, the monovalent L2 immunogens generate neutralizing titers that are greater for the homologous-type virus than for a heterologous-type papillomavirus. The lower immune response to heterologous HPVs could severely limit the breadth and duration of protection of an L2-based vaccine. To address this issue and provide broader immunity, the L2-neutralizing epitope was inserted on the surfaces of VLPs increasing the titers of neutralizing antibodies approximately 10-fold [60]. A synthetic L2 lipopeptide, in which the cross-neutralizing L2 peptide is linked to both a T-helper epitope and a ligand for Toll Like Receptor 2 [TLR2]. tandem repeats of the same peptide displayed on bacterial thioredoxin, or concatenated multitype L2 fusion proteins from different papillomavirus types have already been utilized in inducing cross-neutralizing antibodies against several clinically relevant HPV types[61-63]. In particular, the concatenation of L2 of diverse types results in the repetitive display of B-cell epitopes that enhances antibody production. Indeed, this polymeric L2 approach gives rise to antisera, that neutralize at higher titers, not only the types included in the multimeric immunogen but also other types.

### Low cost vaccines

While the concanated L2 epitope appears to be a promising solution, the VLP/L2 production does not solve the problem of the expensive production of VLPs. Clearly, another drawback in the existing vaccines is that the production of VLPs occurs in eukaryotic cells with high production costs. A cheaper alternative to VLPs is the production of L1 pentamers in bacteria live L1-recombinant *salmonella enterica *serovar typhimurium or typhi that can be stored lyophilized, although multivalent formulations would still be required for broad protection [64-66]. Furthermore, the present vaccine distribution requires a cold chain. This last problem together with high production costs render the wide use of HPV vaccines in near by developing countries almost impossible, where it is most needed because of the lack of cytologic screening programs.

Local production in emerging economies can be the solution, particularly if carried out with the development of very low cost technologies, such as plant-production of the VLP or L2 vaccines [67,68]. A number of studies demonstrated that VLPs from HPVs can also be produced in a variety of plant species including tobacco, potatoes and tomatoes [69-71]. The major drawback in the first few studies was the low production of antigens per gram of the total soluble proteins [TSP]. Recently, by using transient expression technologies, the plant-production of L1 antigens reached 24% [3 g/kg] of the TSP, rendering this preparation useful for industrial scale-up [72]. Finally, VLP preparation can be produced in tomatoes, providing the possibility to deliver inexpensive heat-stable oral vaccines, formulated on site as suspensions to be drunk under supervision [73]. However, the L1 VLPs to date have demonstrated relatively poor immunogenicity when taken orally.

## Conclusion

L1 VLP vaccines are very effective in preventing new infections by the two most common oncogenic HPV types and will dramatically reduce the rates of HPV-associated cancer provided that the vaccine is widely and properly delivered. To reach these conditions more studies are needed in order to find new broad-spectrum vaccines, possibly more economically produced. Furthermore, the above mentioned clinical benefits on the population will emerge only when harmonizing the prevention strategies [primary and secondary] and assuring over time clear and complete information to the community will take place [74]. In addition, the future aim in eradicating HPV-associated pathologies worldwide will be by locally producing antigens with cross-activity among the different types of HPVs.

## Competing interests

The authors declare that they have no competing interests.

## Authors' contributions

LM and AV conceived the study, and participated in its design and coordination and helped to draft the manuscript. All authors read and approved the final manuscript.

## Authors' information

LM is in charge of HPV research at The Dept. of Gynaecologic Oncology - National Cancer Institute Regina Elena of Rome [Italy];

AV is the acting Chief of the Laboratory of Virology - National Cancer Institute Regina Elena of Rome [Italy]
